# Psychometric Validation of the Purpose in Life Test-Short Form (PIL-SF) in Individuals Diagnosed with Severe Mental Illness

**DOI:** 10.3390/healthcare12202082

**Published:** 2024-10-18

**Authors:** César Rubio-Belmonte, Teresa Mayordomo-Rodríguez, Adrià Marco-Ahullo, Inmaculada Aragonés-Barberá

**Affiliations:** Faculty of Psychology, Department of Personality, Treatments and Methodology, Universidad Católica de Valencia “San Vicente Mártir”, 46100 Valencia, Spain; cesar.rubio@ucv.es (C.R.-B.); adria.marco@ucv.es (A.M.-A.); inmaculada.aragones@ucv.es (I.A.-B.)

**Keywords:** PIL-SF, meaning in life, severe mental illness, reliability, structural validity, construct validity

## Abstract

Background: Meaning in Life (MiL) represents a key variable in mental health models of personal recovery. There is a need for straightforward and concise instruments to assess this construct quantitatively in individuals diagnosed with severe mental illness (SMI). Objective: The aim of the present study was to test the psychometric properties of the Purpose in Life Test-Short Form (PIL-SF), a brief self-report measuring the presence of MiL, in a sample of individuals with SMI. Methods: The participants were 41 adults (21 women, 51.8% and 20 men, 48.2%) aged 18 to 65 years (M = 50.05; SD = 10.73) with a diagnosis of SMI (schizophrenia, 61%; bipolar disorder, 26.8%; borderline personality disorder, 7.3%; and major depression, 4.9%) and clinically stable. The PIL-SF, Satisfaction with Life Scale (SWLS), Oxford Happiness Questionnaire—6 Item (OHQ-6), Engagement in Meaningful Activities Survey (EMAS), and Seeking of Noetic Goals—8 Item (SONG-8) were used. Descriptive analysis, estimation of the internal consistency, and Confirmatory Factor Analysis of the PIL-SF were conducted. Furthermore, correlations between the PIL-SF, SWLS, OHQ-6, EMAS, and SONG-8 were calculated. Results: The PIL-SF showed acceptable internal consistency (ω = 0.81) and an excellent fit as a unidimensional scale (CFI = 1.000, TLI = 1.070, RMSEA = 0.000, SRMR = 0.021), confirming its factorial structure. Regarding construct validity, correlations between the PIL-SF and the SWLS (ρ = 0.54, *p* < 0.001), the OHQ-6 (ρ = 0.52, *p* < 0.001), and EMAS (ρ = 0.44, *p* < 0.005) were positive and significant, whereas the correlations between the PIL-SF and the SONG-8NfM (ρ = −0.35, *p* < 0.025) were negative and significant. Conclusions: The Spanish version of the PIL-SF appears to be a reliable and valid instrument to measure the presence of MiL in adults with SMI.

## 1. Introduction

In recent years, a notable shift in the focus of mental health research has occurred. Traditionally, research has been conducted within a deficit-focused and clinical recovery model. However, there is now a growing emphasis on models that focus on the personal recovery of the individual with a mental illness [1,2]. In this sense, the recovery model assigns greater value to the ongoing process of change in personal identity than to the achievement of outcomes related to the disappearance of mental illness symptoms. Similarly, most countries’ mental health policies predominantly follow a recovery-oriented approach. This approach emphasizes the importance of basing services that provide treatment and support to individuals with SMI on the best available evidence and a focus on the recovery process (e.g., HM Government [3]).

Recovery in mental health was defined by Anthony [4] as follows:

“A deeply personal, unique process of changing one’s attitudes, values, feelings, goals, skills, and/or roles. It is a way of living a satisfying, hopeful, and contributing life even within the limitations caused by illness. Recovery involves the development of new meaning and purpose in one’s life as one grows beyond the catastrophic effects of mental illness [4]”.

The recovery model highlights the importance of enabling individuals with severe mental illness to develop a comprehensive life project, focusing on Meaning in Life (MiL) as well as life purposes. Throughout this process, the individual learns to adequately manage the illness to reduce the impact of symptoms and associated disabilities and to achieve valuable goals that extend beyond the label of being ill [5]. In this regard, Heisel and Flett [6] found that suicidal ideation was inversely correlated with both MiL and well-being. This finding highlights the critical role of a lack of goals and a lack of MiL as key factors in suicidal behavior. Given that the lifetime prevalence of completed suicide among individuals diagnosed with schizophrenia is 5.6%, with most incidents occurring during the early stages of the illness [7], it becomes particularly important to explore positive variables within the recovery model.

Within this new paradigm, MiL has attracted increasing interest in both research and clinical intervention. To illustrate this, the CHIME model of personal recovery [8] identifies five principal axes on which to develop personal recovery processes: (1) C, Connectedness; (2) H, Hope and Optimism; (3) I, Identity; (4) M, Meaning; and (5) E, Empowerment. Thus, MiL (M) appears to be an essential axis in the personal recovery process. Leamy et al. [8] identified issues such as ‘meaningful life and social roles’, ‘meaningful life and social goals’, and ‘spirituality’ as relevant to this construct. Furthermore, these authors identified aspects closely related to MiL in the axis related to hope and optimism about the future (H). These include ‘having dreams and aspirations’ (presence of life goals and purposes) or ‘positive thinking and valuing success’ (satisfaction and MiL), among others. Therefore, in the recovery model, the individual’s psychosocial adaptation and the development of a personal project are of primary importance, whereas the difficulties and deficiencies linked to mental illness are considered secondary.

In accordance with this paradigm, which places less emphasis on the role of symptoms, the measurement of the recovery experience has become a priority in the field of clinical mental health [9]. Nevertheless, recovery outcomes have been predominantly evaluated from a subjective perspective through the use of a qualitative methodology [10]. It is essential to introduce quantitative measures that facilitate a more straightforward and time-efficient assessment of patient recovery. This will enable the generation of objective data, allowing for the comparison of results within the same patient (before and after the intervention) and across large samples of subjects with SMI. Thus, there is a need for the development and validation of useful tools with optimal psychometric properties for the quantitative assessment of the presence of MiL and, thus, of aspects closely related to the experience of recovery in individuals with SMI.

### 1.1. MIL and Its Assessment: The Purpose in Life Test

As Steger et al. define it, MiL is “the degree to which people have achieved comprehension (through making sense of their lives and experience, developing a coherent mental model of their selves, the world around them, and their fit and interactions with the world) and have achieved purpose (through discerning, committing to, and pursuing overarching lifelong goals, aims, and aspirations) [11]”.

From the paradigm of humanistic psychology, MiL is recognized as one of the fundamental aspects of understanding well-being [12], it being an essential human motivation [13].

MiL is a critical factor in psychological and spiritual well-being [14]. It is associated with better quality of life [15], better self-perceived health [16], positive feelings and emotions, and a lower incidence of psychological disorders [17,18] and suicidal ideation, even in depressed patients [6].

Given the relevance of MiL to people’s quality of life and well-being, it is important to develop and validate assessment instruments for this construct. Currently, several instruments are used to assess MiL highlighting: the Meaning in Life Questionnaire (MLQ; [19]), with a short version (MLQ-SF; [20]); the Purpose in Life Test (PIL; [21,22]); the Life Attitude Profile (LAP; [23]); or the Life Purpose Questionnaire (LPQ; [24]). Among these, PIL stands out as the most widely used in clinical and nonclinical populations [25,26].

The original version of the PIL comprises 20 items, with responses provided on a 7-point Likert-type scale. Total scores range from 20 to 140, with higher scores indicating a greater perceived Meaning in Life.

Different versions of the PIL have provided adequate psychometric properties in different contexts, such as Argentina [27], Canada [28], Spain [29], Italy [30], and Sweden [31], among others. Despite its wide acceptance in the academic community, PIL has been heavily criticized due to the presence of different underlying factor structures, which limits the suggestion of a consistently replicable model [26].

Prior research has conducted statistical analyses on the original scale with the objective of refining and simplifying it for enhanced usability, resulting in shortened versions that are commonly employed in clinical practice. One such example is the 10-item Spanish version, namely PIL-10 [29], or the shorter version of the PIL, the Purpose in Life-Short Form (PIL-SF; [32]), a four-item unidimensional scale.

### 1.2. The Purpose in Life Test-Short Form (PIL-SF)

The PIL-SF is considered a ‘pure’ measure of meaning and purpose in life [33], in contrast to the original 20-item PIL, which was criticized for including items that were too closely related and for measuring different constructs.

This brief form (items 3, 4, 8, and 20) of the 20-item PIL was developed following several studies [26,32] aimed at determining the factor structure of the PIL via factor analysis procedures. First, Schulenberg and Melton [26] reported an excellent fit of the PIL-SF as a unidimensional scale. Shortly thereafter, Schulenberg et al. [32] extended the evidence of its psychometric properties with the following findings: (1) acceptable internal consistency; (2) adequate fit of the previously obtained unidimensional model; and (3) adequate construct validity on the basis of the positive and significant correlation between the presence of MiL (PIL-SF) and life satisfaction (SWLS, Satisfaction with Life Scale; [34]) and the negative and significant correlation between the presence of MiL (PIL-SF) and the Search for Meaning (SONG, Seeking of Noetic Goals; [35]).

The PIL-SF has been translated and adapted in several contexts, including Denmark [36], Argentina [37], Ecuador [38], China [39], and Spain [40]. These and other studies with a U.S. population (e.g., Descher [41]; Schulenberg et al. [32]) have provided strong evidence for the psychometric validity of this scale (reliability and construct validity), as well as for the structural validity of the PIL-SF model as a unidimensional scale (see Table S1). However, most of these studies were conducted with nonclinical samples of adolescents (e.g., Schulenberg et al. [33]), undergraduates/emerging adults (e.g., Schnetzer et al. [42]), or adults (e.g., Caycho-Rodriguez et al. [43]).

In studies involving clinical populations, only the study by Peter et al. [44] with individuals who have spinal cord injuries has shown results regarding internal consistency and concurrent validity. Studies by Cheraghifard et al. [45,46] with stroke survivors have only shown results on concurrent validity. None of the studies with clinical samples have tested the factor structure of the instrument in the populations analyzed.

The Purpose in Life Test-Short Form (PIL-SF) is a brief self-report measure of the presence of MiL, more specifically, the presence of meaning (PoM), which has not previously been validated in individuals with SMI. Thus, the interest in variables related to MiL in models of personal recovery in SMI, together with the need to have validated instruments for measuring such variables in this clinical population, justifies the interest of the present study.

The aim of the present study was to test the factor structure of the PIL-SF and analyze its psychometric properties in a sample of individuals with severe mental illness.

The aforementioned aims were operationalized into the following specific objectives: (1) to confirm the factorial structure of the PIL-SF through confirmatory factor analysis procedures; (2) to analyze the reliability of the PIL-SF by estimating its internal consistency; and (3) to verify the construct validity of the PIL-SF by examining its correlation with other measures of personal well-being, MiL, and occupational engagement.

## 2. Materials and Methods

### 2.1. Participants

Forty-one adults (21 women, 51.8% and 20 men, 48.2%), users of a care device for people with SMI aged 18 to 65 years (M = 50.05; SD = 10.73) with a diagnosis of severe mental illness (25 with schizophrenia, 61%; 11 with bipolar disorder, 26.8%; 3 with borderline personality disorder, 7.3%; and 2 with major depression, 4.9%). Sampling was performed using convenience sampling. The participants were required to meet the criteria for clinical stability, defined as no pharmacological changes in the past 6 months or hospital admissions in the past 12 months. Informed consent was obtained from all participants, who participated voluntarily and anonymously and received no compensation for their participation.

The sample of the present study consisted exclusively of individuals with a diagnosis of severe mental illness (SMI) who were users of community rehabilitation services in the province of Valencia (Spain), which ensured a high degree of uniformity in the sample context. Data collection was carried out between January and May 2024 by a member of the research team accompanied by a rehabilitation resource professional.

### 2.2. Instruments

Purpose In Life-Short Form (PIL-SF; [32]). The PIL-SF is an abbreviated adaptation of the Crumbaugh and Maholic [22] scale for assessing the presence of MiL. The Spanish version of Rubio-Belmonte et al. [40] was used. The PIL-SF consists of 4 items assessing satisfaction with life and purpose and goals in life. The total score ranges from 4 to 28. The higher the PIL-SF score, the greater the MiL.

Satisfaction With Life Scale (SWLS; [34]). The Spanish adaptation of the SWLS by Arce [47] was used. The SWLS consists of 5 items, scored from 1 (strongly disagree) to 7 (strongly agree), which assess global cognitive judgments of life satisfaction. The total score ranges from 5 to 35. The higher the score, the greater the life satisfaction. In the present study, the SWLS showed good internal consistency, ω = 0.80.

Oxford Happiness Questionnaire—6 Item (OHQ-6; [48]). The OHQ-6 is a shortened, adapted, and validated version of the Oxford Happiness Questionnaire—8 items [49] in people with SMI that assesses happiness. This self-report questionnaire consists of 6 items scored on a Likert scale (1 = strongly disagree; 6 = strongly agree). The score ranges from 6 to 36. The higher the OHQ-6 score, the greater the level of happiness. In the present study, the OHQ-6 showed good internal consistency, ω = 0.78.

Engagement in Meaningful Activities Survey (EMAS; [50]). The Spanish adaptation of the EMAS by Fernández-Solano et al. [51] was used. The EMAS consists of 12 items scored on a Likert scale from 1 (never) to 5 (always), which assess the extent of a person’s engagement in meaningful and fulfilling occupations. The maximum score is 60. The higher the score, the greater the attribution of meaning to daily activities. In the present study, the EMAS showed good internal consistency, ω = 0.80.

Seeking of Noetic Goals—8 Item (SONG-8; [52]). This Spanish version of the Seeking of Noetic Goals (SONGs; [35]) assesses the motivational intensity of Search for Meaning (SfM) in life with an 8-item Likert scale (1 = never; 7 = always). It has a two-factor structure: Need for Meaning (NfM) and Expectations (EX). The total score ranges from 8 to 56. A high score is indicative of high motivation for Search for Meaning (SfM). In this study, the SONG-8 demonstrated acceptable internal consistency, with a McDonald´s omega coefficient of ω = 0.74.

### 2.3. Procedure

The study authors obtained permission from the clinical managers of the institution where the participants received support and care in order to recruit the sample. Subsequently, the subjects were invited to participate in the research on a voluntary and unpaid basis. Individual informed consent was obtained from all study participants. The participants completed the questionnaire protocol with the support and supervision of one of the authors of this study, who briefly explained the nature and aims of the study in a manner that did not emphasize aspects that might bias the responses. Any doubts about the procedure were clarified, and the participants were guaranteed anonymity and confidentiality. The authors requested honest responses to maximize the validity of the data.

### 2.4. Statistical Analyses

The initial step involved a descriptive analysis and internal consistency assessment of the PIL-SF, with the objective of evaluating the data distribution and assessing the reliability of the instrument. Given that the scales employed in this study are ordinal and that Cronbach’s alpha is known to underestimate the internal consistency of such scales [53], the more robust McDonald’s omega (ω) [54] was used to estimate the internal consistency of these scales. This index has a value between 0 and 1, and internal consistency is deemed acceptable if ω is equal to or greater than 0.70 [55].

Second, a Confirmatory Factor Analysis (CFA) of the PIL-SF was conducted. Given that the scale is ordinal and taking into account Mardia’s coefficient (9.29), it was not possible to assume multivariate normality [56]. Consequently, the diagonally weighted least squares (DWLSs) method with robust estimation was employed [57]. The fit indices included the comparative fit index (CFI; [58]) and the Tucker–Lewis index (TLI; [59]). An acceptable fit was indicated by a value of ≥0.90, whereas a good model fit was indicated by a value of ≥0.95. Additionally, the RMSEA (root mean square error of approximation) and the SRMR (standardized root mean square residual) were considered [60]. Values of ≤0.08 indicated an acceptable model fit, whereas values of ≤0.05 indicated a good model fit (e.g., Hair et al. [61]).

Finally, to test the concurrent validity of the PIL-SF, correlations were analyzed with the SWLS [47], OHQ-6 [48], EMAS [51], and SONG-8 [52]. The SWLS and OHQ-6 were selected for the analysis of convergent validity, as they assess constructs pertaining to well-being, life satisfaction, and happiness, respectively. Similarly, the EMAS was selected for the analysis of convergent validity, as it assesses factors pertaining to meaning, engagement, and achievement in relation to involvement in activities. Finally, the SONG-8 was employed for the analysis of divergent validity due to its complementary nature in relation to the PIL-SF [62,63], which is a measure of SfM. Pearson correlations were employed for these analyses. The effect sizes of the correlations were interpreted in accordance with the criteria established by Cohen [64]. Effect sizes between 0.20 and 0.49 represented a weak effect, those between 0.50 and 0.79 a moderate effect, and those of 0.80 or above a strong effect.

All the statistical analyses were conducted via the open-source software JASP 0.18.3 for Windows [65].

## 3. Results

### 3.1. Descriptive Statistics and Internal Consistency of the PIL-SF

The mean and standard deviation of the PIL-SF total score were M = 20.95 and SD = 5.62, respectively. The PIL-SF showed acceptable internal consistency, ω = 0.81 (which did not improve with the removal of any item), and an average interitem correlation of 0.50. The corrected item-total correlations were moderate (ranging from 0.49 to 0.67) (Table 1).

Acceptable internal consistency was found for the other scales used in this study: SWLS, ω = 0.80; OHQ-6, ω = 0.78; EMAS, ω = 0.80; and SONG-8, ω = 0.74.

### 3.2. Structural Validity of the PIL-SF

**Figure 1:**
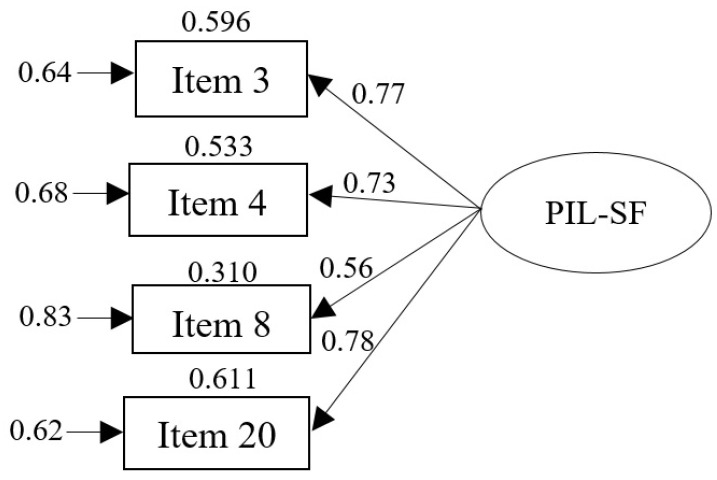
Standardized solution of the PIL-SF. The values on the right indicate the standardized regression coefficients. The values on the left of each item of the model are errors. The values above each item represent R^2^.

A Confirmatory Factor Analysis (CFA) was conducted for the PIL-SF, yielding a Mardia’s normalized estimate coefficient of 9.29. The proposed unidimensional model demonstrated an excellent fit: χ^2^(6) = 51.107, *p* = 0.620, CFI = 1.000, TLI = 1.070, RMSEA = 0.000 (90% CI [0.000, 0.252], SRMR = 0.021 (Figure 1). The estimated standardized parameters were significant (*p* < 0.05) [66]. However, it should be noted that item 8 does not reach optimal values (r > 0.70) [67].

### 3.3. Construct Validity of the PIL-SF

The PIL-SF score was positively correlated with the SWLS score (ρ = 0.54, *p* < 0.001), with a moderate effect size. Similarly, a positive correlation was observed between the PIL-SF and the OHQ-6 (ρ = 0.52, *p* < 0.001), also with a moderate effect size. Furthermore, a positive correlation was identified between the PIL-SF and the EMAS (ρ = 0.44, *p* < 0.005), with a moderate effect size. Conversely, a negative correlation was observed between the PIL-SF and the SONG-8NfM (ρ = −0.35, *p* < 0.025), with an effect size considered weak. The PIL-SF showed significant correlations in the hypothesized direction, which is consistent with the intended assessment of the scales.

## 4. Discussion

The aim of the present study was to examine the psychometric properties of the PIL-SF in a sample of adults diagnosed with SMI. The following hypotheses were tested: (1) the PIL-SF has good reliability, as indicated by internal consistency; (2) the PIL-SF would exhibit a good fit with a unidimensional structure; and (3) the PIL-SF has good construct validity.

The Spanish validation of the PIL-SF for individuals diagnosed with SMI demonstrated good internal consistency and structural validity. The presence of MiL was found to be positively correlated with life satisfaction, happiness, and engagement in meaningful and fulfilling occupations and negatively correlated with SfM, specifically with the NfM. The results demonstrate that the PIL-SF is a reliable and valid instrument for assessing the presence of MiL in individuals with SMI.

### 4.1. Descriptive Statistics and Internal Consistency of the PIL-SF

To the best of our knowledge, this study represents the first validation of the PIL-SF with individuals diagnosed with SMI. The PIL-SF has been employed in a selection of published studies with individuals diagnosed with neurological injuries such as Spinal Cord Injury (SCI) [44] and stroke survivors [45,46]. However, the two aforementioned studies are related to the psychometric validation of other instruments (MAPA and EMAS, respectively). In both cases, the PIL-SF was employed to assess convergent validity, and there is a paucity of psychometric data available on the PIL-SF in such studies with clinical samples.

The mean score obtained in the present study was 20.95 (SD = 5.62), with scores ranging from 4 to 28. These findings are comparable to those of the Spanish validation study with a nonclinical sample (M = 22.77, SD = 3.53) by Rubio-Belmonte et al. [40] and to the results reported by Peter et al. [44] in their study with SCI people (M = 21.3, SD = 4.6).

In terms of internal consistency, the PIL-SF demonstrated an acceptable McDonald’s ω of 0.81, given the limited number of items in the instrument [68]. This result is comparable to that of other validation and adaptation studies, with consistency indices yielding values of approximately 0.80, as observed in studies involving non-Spanish (e.g., Pacak-Vedel et al. [36]; Schnetzer et al. [42]; Weber et al. [37]; Xiao & Li [39]) and Spanish samples [40]. The results of the aforementioned studies demonstrate that this adaptation has satisfactory internal consistency and reliability in this particular sample. Furthermore, the item-total correlations indicate that the PIL-SF items contribute to the scale’s reliability by measuring the same construct, namely, the presence of MiL.

### 4.2. Structural Validity of the PIL-SF

The unidimensional model proposed by Schulenberg and Melton [26] demonstrated an adequate fit in the present study, confirming the appropriateness of the 4-item model (items 3, 4, 8, and 20) for use in individuals diagnosed with SMI. The fit indices obtained are consistent with the findings of other studies that have employed CFA procedures to test the factor structure of the PIL-SF in adult samples (e.g., Caycho-Rodríguez et al. [43]; Pacak-Vedel et al. [36]), emerging adults/undergraduates (e.g., Schulenberg et al. [32]; Weber et al. [37]), and adolescents (e.g., Schulenberg et al. [33]), indicating that the structure identified by Schulenberg and Melton [26] can be replicated. To the best of our knowledge, no other studies have verified the factor structure of the PIL-SF with clinical samples, particularly those with SMI.

Despite the aforementioned evidence, it should be noted that the item PIL-SF8 exhibited a low factor loading (0.56) in the CFA. This finding is similar to that obtained in other studies in which the PIL-SF8 item has a factor loading below 0.70 (e.g., Moreta-Herrera et al. [38]; Rubio-Belmonte et al. [40]; Weber et al. [37]). The PIL-SF8 statement item is as follows: ‘In achieving life goals I have made no progress whatever/progressed to complete fulfillment’, so the item’s content seems to focus on a retrospective evaluation of ‘Life goal completion’. In contrast, the remaining items’ contents are aimed at making a judgment of MiL in the present moment or even prospectively: PIL-SF3, ‘Presence of clear life goals’; PIL-SF4, ‘Life being meaningful’; and PIL-SF20, ‘Presence of goals/life purposes’. It can be posited that the low factor loading of the item is a consequence of the characteristics of the sample used in this study. Thus, participants with a diagnosis of SMI, particularly those who have had a chronic condition for many years, may perceive that they have not made significant progress toward achieving their life goals, and this, therefore, makes it difficult to assess their own level of self-fulfillment. It seems reasonable to suggest that the PIL-SF8 item is not merely age-sensitive, as previously asserted by Rubio-Belmonte et al. [40], but may also be clinically sensitive. Future studies using clinical samples should investigate these findings further.

### 4.3. Construct Validity of the PIL-SF

Significant positive correlations were identified between Meaning in Life (PIL-SF) and both life satisfaction (SWLS, ρ = 0.54) and happiness (OHQ-6, ρ = 0.52). Regarding the relationships between PIL-SF and SWLS, the values obtained in the present study concur with the findings of previous research conducted by Schulenberg et al. [31], Rubio-Belmonte et al. [39], and Peter et al. [43] with SCI individuals, and exceed the values reported by Drescher et al. [40], Schulenberg et al. [32], Weber et al. [37], and Xiao and Li [39]. Concerning the relationship between meaningfulness and happiness, the correlation value between the PIL-SF and OHQ-6 obtained in the present study is similar to those previously reported by Rubio-Belmonte et al. [40] with the OHQ-8 and higher than those reported by Pacak-Vedel et al. [36] with the Well-Being Index [69]. These findings contribute to the growing body of evidence supporting the notion that there is a clear positive correlation between MiL and life satisfaction and subjective well-being. This finding aligns with the general consensus that MiL is beneficial for overall well-being and a fulfilling life (e.g., Van Tilburg and Igou [70]).

A significant positive correlation was observed between engagement in meaningful activities (EMAS) and PIL-SF scores (ρ = 0.44). The correlations between the PIL-SF and EMAS were lower than those previously reported by Cheraguifard et al. [45] and similar to those reported by Cheraguifard et al. [46], who used the PIL-SF and the Meaningful Activities Participation Assessment (MAPA; Eakman et al. [71]) with stroke survivors. Participation in meaningful activities contributes to individual well-being and the satisfaction of individuals’ psychological, biological, and cultural needs for a meaningful life, thus improving their emotional, cognitive, and physical states (e.g., Goldberg et al. [50]; Han et al. [72]; Plow et al. [73].

A significant negative correlation was observed between the PIL-SF and the SONG-8NfM (ρ = −0.35). This result is greater than that obtained by Rubio-Belmonte et al. [40] and Shulenberg et al. [32] with the PIL-SF and the extended 20-item version of the SONG [34], thus reinforcing the complementary nature of PIL-SF [39] and SONG-8 [51]. This negative correlation indicates that an increase in the presence of MiL is associated with a decrease in the motivation for SfM and purpose in life. Conversely, an individual who lacks Meaning in Life may be motivated to Search for Meaning to satisfy this need (e.g., Park and Folkman [73]).

In conclusion, the greater the presence of MiL, the greater the satisfaction with life, the greater the happiness, the greater the commitment to meaningful occupations, and the lesser the need to SfM. On the basis of these results, the PIL-SF is a valid scale to measure variables related to MiL.

### 4.4. Study Limitations and Directions for Future Research

It is important to recognize the limitations of this study and identify potential avenues for future research based on the presented findings.

The sampling method and sample composition restrict the extent to which the results can be generalized. Although the number of participants is adequate for the proposed statistical analyses, future studies should employ larger random samples that are balanced with regard to SMI diagnoses. This will ensure the generalizability of the results and the statistical robustness of the comparisons and provide the opportunity to conduct supplementary psychometric analyses, such as those examining the invariance of age and gender.

The cross-sectional design of this study represents a limitation in terms of the possibility of examining test-retest reliability and the study of causal relationships between the variables under study, which are scarce in the current literature. Further studies with a longitudinal and cross-cultural design could extend the results of the temporal reliability of this scale and of causal relationships between variables of interest in recovery models, such as the presence of MiL, the SfM, subjective well-being, or engagement in meaningful occupations. The decision to use a cross-sectional design in this research was driven by time constraints on access to the study population, which made a longitudinal design impractical. However, the cross-sectional approach effectively addressed the objectives of this study and provided an appropriate strategy for maximizing the collection of relevant information, given the limitations of sample availability.

Future research should examine the psychometric properties of the PIL-SF in different populations (e.g., older adults, caregivers of dependent persons, individuals with disabilities or chronic and terminal illnesses). It would be appropriate to include other measures of MiL (e.g., MLQ, Steger et al. [19]) to extend the evidence of construct validity. As noted by Schulenberg and Melton [26], having a concise instrument with strong psychometric properties for assessing MiL is of great value.

In addition to the aforementioned limitations, the present study offers several noteworthy contributions. The PIL-SF is a brief, self-administered scale for measuring MiL that exhibits optimal psychometric properties in individuals diagnosed with SMI. To our knowledge, this study is pioneering in exploring the factor structure of the PIL-SF in a clinical sample and, more specifically, in individuals diagnosed with SMI. Notably, the American Psychological Association has issued guidelines recommending that psychological instruments should undergo validation and testing in diverse samples, particularly when they are employed in populations that may differ from those in which the test was originally developed [74]. Furthermore, the APA’s guidelines on evidence-based psychological practice in healthcare [75] advise that assessments should be appropriate for the setting, purpose, and population.

The availability of a brief and psychometrically robust measure of the presence of MIL in individuals with an SMI diagnosis provides opportunities for clinical and research settings. Thus, the use of the PIL-SF may reduce the burden of assessment on patients while simultaneously enabling professionals to assess a construct that provides pertinent information regarding the recovery process of individuals with SMI.

The use of the PIL-SF in clinical settings presents a variety of potential benefits, including the following: (1) less time and effort to respond, thereby enhancing its potential usefulness in populations that have difficulty completing questionnaires; (2) a cost-effective method for the assessment of individuals with SMI in Public Health settings; and (3) easier routine monitoring of outcomes throughout the therapeutic process (e.g., between sessions), particularly for patients who do not respond as expected to the therapeutic intervention [76]. Future research with individuals diagnosed with SMI may contribute to validating the clinical utility of the PIL-SF as a measure of MiL, a central construct of the recovery model, and its sensitivity to therapeutic change.

The World Health Organization, through its QualityRights initiative, has reinforced its commitment to improving mental health services by promoting the recovery of individuals with severe mental illness (SMI) [77]. Given that clinical recovery and personal recovery are distinct constructs that should be assessed separately [78], and considering the four key components identified by Andresen et al. [10] in the recovery model—(1) finding hope, (2) identity, (3) meaning in life, and (4) taking responsibility—it is evident that having a validated version of the PIL-SF for this population presents an opportunity to measure MiL and monitor this crucial variable throughout the personal recovery process. This could have a positive impact on the quality of life and well-being of individuals with SMI. In particular, the PIL-SF could be a valuable tool for evaluating the final stage of Andresen et al.‘s recovery model [10], known as ‘Growth’. This stage assesses whether an individual has achieved a positive sense of self, a purposeful and fulfilling life, and a hopeful vision for the future.

With regard to the use of the PIL-SF and other brief measures in a research setting, the following points may be considered: (1) the reduction in response time may favor the development of longitudinal studies or large assessment protocols with a broad spectrum of variables, and (2) the use of the PIL-SF may facilitate momentary and real-time ecological assessment as an alternative to retrospective self-reports [79].

## 5. Conclusions

To the best of our knowledge, this is the first study to address the validation of the PIL-SF in individuals with SMI. The Spanish version of the PIL-SF appears to be a reliable and valid instrument for measuring MiL in adults with SMI. The use of brief scales with excellent psychometric properties facilitates the evaluation of clinical samples, such as the one in the present study. Further research is needed to expand the evidence for the psychometric properties of the PIL-SF and to validate this scale in other clinical samples.

## Figures and Tables

**Table 1:** Descriptive statistics of the scales used in the present study.

Items of the PIL-SF and Scales		M	SD	Skewness (SE)	Kurtosis (SE)	*r* Item-Test	ω If Item Dropped
PIL-SF3	*In life I have one of the following: no goals or aims/clear goals and aims./*En la vida tengo: ninguna meta o anhelo/muchas metas y anhelos definidos.	5.10	2.02	−0.86 (0.37)	−0.70 (0.72)	0.65 *	0.73
PIL-SF4	*My personal existence is one of the following: utterly meaningless, without purpose/purposeful, and meaningful./*Mi existencia personal es: sin sentido ni propósito/llena de sentidos y propósito.	5.42	1.85	−1.22 (0.37)	0.37 (0.72)	0.64 *	0.74
PIL-SF8	*In achieving life goals I have made no progress whatever/progressed to complete fulfilment.*/En el logro de mis metas vitales: no he conseguido ningún progreso/he llegado a mi realización completa.	5.17	1.56	−1.25 (0.37)	1.31 (0.72)	0.49 *	0.80
PIL-SF20	*I have discovered one of the following: no mission or purpose in life/a satisfying life purpose.*/He descubierto: ninguna misión o propósito en mi vida/metas claras y un propósito satisfactorio para mi vida.	5.39	1.53	−1.19 (0.37)	1.03 (0.72)	0.67 *	0.74
PIL-SF		20.95	5.62	−0.97 (0.37)	0.34 (0.72)		
SWLS		21.10	7.05	−0.18 (0.37)	−1.16 (0.72)		
OHQ-6		26.90	6.43	−0.68 (0.37)	0.24 (0.72)		
EMAS		38.10	6.16	−0.43 (0.37)	−0.60 (0.72)		
SONG-8NfM		20.29	3.74	−0.46 (0.37)	−1.06 (0.72)		
SONG-8EX		16.29	6.43	−0.68 (0.37)	0.24 (0.72)		

Note. *n* = 41. The numbering of the items of the original version was preserved. In italics, the Crumbaugh and Maholick’s (1964, 1969) original items. In normal, the Spanish translation of the items. SE = Standard Error; PIL-SF = Purpose in Life Test-Short Form; SWLS = Satisfaction With Life Scale; OHQ-6 = Oxford Happiness Questionnaire—6 Item; EMAS = Engagement in Meaningful Activities Survey; SONG-8 = Seeking of Noetic Goals—8 Item; NfM = Need for Meaning; EX = Expectations.; * *p* < 0.001.

## Data Availability

The data presented in this study are available on request from the corresponding author.

## Supplementary Materials

The following supporting information can be downloaded at https://www.mdpi.com/article/10.3390/healthcare12202082/s1. Table S1. Studies that have analyzed the psychometric properties of the PIL-SF. Ref. [80] is cited in the supplementary materials.
