# Supplementary material for: Psychometric Validation of the Purpose in Life Test-Short Form (PIL-SF) in Individuals Diagnosed with Severe Mental Illness
Source: Healthcare (Basel). 2024 Oct 18;12(20):2082. doi: 10.3390/healthcare12202082 (PMC11507619; doi:10.3390/healthcare12202082)
Supplement: Supplementary file 1 [file healthcare-12-02082-s001.zip › healthcare-3241969-supplementary.pdf]

Table S1

Studies that have analyzed the psychometric properties of the PIL-SF

Authors (year)	Sample		α	ϖ	Descriptive	Concurrent validity			Structural validity				
	N	Age: *Mean(SD)*			*M/SD*	SWLS	SONG	Others	$\chi^2(2)$	CFI	TLI	RMSEA	RMSR
Non clinical samples													
Caycho-Rodríguez et al. (2023)	4306 adults												
	Argentinians	36.4(15.3)	.85	.85	NR				17.11	.99	.99	.075 [.045, .109]	.012
	Colombians	32.9(12)	.87	.87	NR				2.66	1.00	.99	.032 [.000, .121]	.007
	Ecuadorians	24.6(7.8)	.86	.85	NR				.86	1.00	1.00	.000 [.000, .056]	.003
	Salavadorans	28.7(8.8)	.87	.87	NR				1.07	1.00	1.00	.000 [.000, .094]	.006
	Mexicans	34.6 (11.6)	.86	.86	NR				1.37	1.00	1.00	.000 [.000, .059]	.005
	Paraguayans	36.9(11.5)	.84	.83	NR				.50	1.00	1.00	.000 [.000, .083]	.005
	Uruguayans	41.8(12.6)	.86	.88	NR				2.14	1.00	1.00	.013 [.000, .101]	.010
Drescher et al. (2012)	361 American adults	38.18(13.20)	.86	NR	19.32/ 5.47	.50							
Moreta-Herrera et al. (2023)	743 Ecuadorians adults	24,7(7.9)	.85	NR	NR			-.43[2]		.999	.998	.001 [.000, .005]	.003
Pacak-Vedel et al. (2021)	4849 Danish adults	41.7(12.16)	.79	NR	23.06/3.24			.44[3]	7.14	.999	.997	.23 [.006, .042]	.061
Rubio-Belmonte et al. (2022)	349 Spanish undergraduates	20.85(2.16)	NR	.83	22.77/3.53	.59	-.23	.56[4]	4.26	1.00	.99	.57 [.000,.133]	
Schnetzer et al. (2012)	267 American students	19.14(3.73)	.79	NR	23.33/3.06			-.39[5]					
Schulenberg & Melton (2010)	620 American undergraduates	Sample 1: 19.5(1.6)<br>Sample 2: 19.8(2.6)	NR	NR	NR<br>NR				.30	1.00	1.01	.00 [.00, .06]	
Schulenberg et al. (2011)	298 American undergraduates	19.67(2.27)	.84	NR	22.67/3.73	.53	-.22	-.20[6]<br>.64[7]<br>.58[8]	.06	1.00	1.01	.00 [.00, .02]	
Schulenberg et al. (2016)	91 American adolescents	14.89(NE)	.89	NR	21.34/5.41	.45							
Weber et al. (2022)	704 Argentinians undergratuates	Sample 1: 23.88(3.57)	NR	.79	20.91/4.68	.45			2.01	1.00	1.00	.004 [.001, .103]	.11

		Sample 2: 25.09(5.97)											
Xiao & Li (2016)	997 Chinese college students	NE	.81	NR	NR	.46							
Xiao et al. (2021)	2065 Chinese college students	20.85(1.30)	.86	NR	NR								
Zhu et al. (2021)	1735 Chinese students	20.65(1.34)	.92	NR	18.93/4.76								
Clinical samples													
Cheraghifard et al. (2020)	107 Iranian adults with stroke	NE	NR	NR	*18.79/6.42*			.60[9]<br>.54[10]<br>.31[11]					
Cheraguifard et al. (2022)	123 Iranian adults with stroke	61.26(12.15)	NR	NR	NR			.86[12]					
Peter et al. (2014)	516 Swiss SCI people	53.1(14.6)	.89	NR	21.3/4.6	.58[1]		.55[13]<br>.48[14]					
Current study	***41 Spanish SMI people***	***50.05(10.73)***	***NR***	***.81***	***20.95/5.62***	***.54***	***-.35***	***.52[15]<br>.44[12]***	***51.10***	***1.00***	***1.07***	***.000 [.000, .252]***	***.021***

Note: SCI = Spinal Cord Injury; SMI = Severe Mental Illness; NE = Not specified; NR = Not reported; SWL = Satisfaction With Life; SONG = Seeking Of Noetic Goals; (1) WHO-QOL BREF selected items; (2) PHQ-9 (Patient Health Questionnarie-9 Item; (3) WHO5 (hedonic well-being); (4) OHQ (Oxford Happiness Questionnaire); (5) CES-D (Center for Epidemiological Studies – Depression scale); (6) MLQ-S (Meaning in Life Questionnaire-Search); (7) MLQ-P (Meaning in Life Questionnaire-Presence); (8) LPQ (Life Purpose Questionnaire); (9) MAPA (Meaningful Activity Participation Assessment); (10)MAPA-F (Frecuency); (11) MAPA-M (Meaning); (12) EMAS (Engagement in Meaningful Activities Survey); (13) GSES (General Self-Efficacy); (14) Brief COPE.
